# Calpain system is altered in survival motor neuron-reduced cells from in vitro and in vivo spinal muscular atrophy models

**DOI:** 10.1038/s41419-020-2688-5

**Published:** 2020-06-25

**Authors:** Sandra de la Fuente, Alba Sansa, Iván Hidalgo, Nuria Vivancos, Ricardo Romero-Guevara, Ana Garcera, Rosa M. Soler

**Affiliations:** 1 Neuronal Signaling Unit, Experimental Medicine Department, Universitat de Lleida-IRBLleida, Rovira Roure, 80, Lleida, 25198 Spain; 2 Metabolic Physiopathology Group, Experimental Medicine Department, Universitat de Lleida-IRBLleida, Lleida, Spain

**Keywords:** Neurodegeneration, Motor neuron disease

## Abstract

Spinal muscular atrophy (SMA) is a severe neuromuscular disorder caused by loss of the *survival motor neuron 1* (*SMN1*) gene. SMA is characterized by the degeneration of spinal cord motoneurons (MNs), progressive skeletal muscle atrophy, and weakness. The cellular and molecular mechanisms causing MN loss of function are only partially known. Recent advances in SMA research postulate the role of calpain protease regulating survival motor neuron (SMN) protein and the positive effect on SMA phenotype of treatment with calpain inhibitors. We analyzed the level of calpain pathway members in mice and human cellular SMA models. Results indicate an increase of calpain activity in SMN-reduced MNs. Spinal cord analysis of SMA mice treated with calpeptin, a calpain inhibitor, showed an increase of SMN, calpain, and its endogenous inhibitor calpastatin in MNs. Finally, in vitro calpeptin treatment prevented microtubule-associated protein 1A/1B-light chain 3 (LC3) increase in MNs neurites, indicating that calpain inhibition may reduce autophagosome accumulation in neuron prolongations, but not in soma. Thus, our results show that calpain activity is increased in SMA MNs and its inhibition may have a beneficial effect on SMA phenotype through the increase of SMN in spinal cord MNs.

## Introduction

Survival motor neuron (SMN) is a ubiquitously expressed protein located in the cytoplasm and the nucleus of the cells, where it accumulates in Cajal bodies and Gems (Gemini of Cajal bodies)^1^. SMN complex participates in the assembly of small nuclear ribonuclear proteins (snRNPs), which are involved in the splicing of pre-mRNA^2^. SMN also accomplishes unique functions in motoneurons (MNs), such as the transport of β-actin mRNA to the growth cones of the axons^3^. SMN protein deficiency is the basis of the neuromuscular disease spinal muscular atrophy (SMA). SMA is a devastating disorder that leads to progressive muscle weakness and atrophy and represents the most common lethal genetic disease in infants^4–6^. SMA is characterized by the degeneration of spinal cord MNs caused by the mutation of the *survival motor neuron* (*SMN1*) gene located at the telomeric region of chromosome 5q3, which is responsible for the production of SMN protein^7^. *SMN2* gene, a nearly identical paralog of *SMN1* in centromeric region of chromosome 5q3, is present in the human genome as part of an inverted duplication. *SMN2* expresses limited amounts of functional full-length SMN protein. In SMA, *SMN2* is efficiently transcribed but cannot fully compensate for the loss of *SMN1* because a translationally silent, single-nucleotide transition at position 6 in exon 7 causes predominant exon 7 skipping and results in an unstable protein (SMNdelta7)^8,9^. The number of *SMN2* gene copies inversely correlates with the severity of SMA phenotype and determines disease onset^10^. Although the genetic causes of SMA are well-known, the mechanisms underlying lower MN degeneration remain unclear. For example, MNs may have a uniquely high demand for efficient messenger RNA splicing or disruption of a specific SMN function in MNs may cause SMA^11^. Knowledge of the molecular and cellular mechanisms of MN degeneration could be extremely relevant to prevention and/or delay of SMA progression and to optimization of recently developed therapies^12,13^.

The calpain protease system and autophagy are two major regulatory pathways of the cell that have been correlated to neurodegenerative disorders^14,15^. Calpains are a calcium-dependent family of proteases that participate in calcium-mediated signaling pathways involved in cell processes. Increases of cytosolic calcium in neurons activate calpains and pathological conditions often result in their overactivation^16–18^. Autophagy is a regulated process responsible for the degradation of cytoplasmic proteins and organelles by incorporating them into a double-membrane vesicle (autophagosome) that is delivered to the lysosome^19^. In SMA, autophagy is likely involved in MN death^20^.

Evidence exists that calpains exert modulatory effects at multiple levels of autophagy^14^. In this context, we recently reported that calpain inhibition increases the SMN protein level in cultured spinal cord MNs and that administration of calpeptin (a well-known calpain inhibitor) to SMA mice models improves lifespan and motor function^21^. Based on these results, the aim of the present work was to investigate the calpain pathway in SMN-reduced MNs. We examined calpain activation by monitoring α-fodrin fragments and concluded that calpain activation is increased in cultured human and mice SMA MNs, but not in SMA fibroblasts. In vivo calpeptin treatment increases Smn, calpain, and calpastatin protein levels in spinal cord MNs. Finally, the addition of calpeptin to the culture medium modulated the level of the LC3 autophagy marker in cultured MNs. Our findings suggest that calpain can be overactivated in SMN-reduced MNs and support the hypothesis that calpeptin may be useful in SMA therapies.

## Materials and methods

### SMA animals

Experiments involved two severe SMA mouse models. FVB·Cg-Tg (SMN2)^89Ahmb^Smn1^tm1Msd^/J (mutSMA) and FVB·Cg-*Grm 7*^Tg(SMN2)89Ahmb^ Smn 1^tm1Msd^ Tg(SMN2*delta7)4299Ahmb/J (SMNdelta7) mice were kindly provided by Dr Josep E Esquerda and Dr Jordi Caldero (IRBLleida-Universitat de Lleida). MutSMA (Smn^−/−^; SMN2^+/+^) and SMNdelta7 (Smn^−/−^; SMN2^+/+^; SMNΔ7^+/+^) were obtained by crossing heterozygous animals. Littermates mutSMA/SMNdelta7 and WT (Smn^+/+^; SMN2^+/+^ and Smn^+/+^; SMN2^+/+^; SMNΔ7 ^+/+^, respectively) were used for the experiments.

For MN purification, the heads of 12.5-day embryos (E12.5) were snipped for genotyping; for in vivo treatment, neonatal offspring were tattooed and a fragment of the tail was obtained. The REDExtract-N-Amp Tissue PCR Kit (Sigma) was used for genomic DNA extraction and PCR setup, with the following primers: WT forward 5′-CTCCGGGATATTGGGATTG-3′, SMA reverse 5′-GGTAACGCCAGGGTTTTCC-3′ and WT reverse 5′-TTTCTTCTGGCTGTGCCTTT-3′. All procedures were done in accordance with the Spanish Council on Animal Care guidelines and approved by the Universitat de Lleida Advisory Committee on Animal Services (Comité Ético de Experimentación Animal CEEA 02-01/17).

### Spinal cord MN isolation and culture

MN primary cultures were obtained from the spinal cords of CD1 or WT and mutant SMA mouse embryos at E12.5 essentially as described^22,23^. Isolated cells (approximately 25,000 cells per spinal cord) were pooled in culture medium and plated either in 4-well tissue culture dishes (Nunc, Thermo Fisher Scientific) for western blot analysis (50,000 cells/well) or using 15-mm glass coverslips placed into the 4-well dishes for immunofluorescence experiments (15,000 cells/well). Culture medium was Neurobasal medium (Gibco, Thermo Fisher Scientific) supplemented with B27 (2% v/v; Gibco), horse serum (2% v/v; Gibco), l-glutamine (0.5 mM; Gibco) and 2-mercaptoethanol (25 μM; Sigma) and a cocktail of brain-derived neurotrophic factor (BDNF), glial cell line-derived neurotrophic factor (GDNF), ciliary neurotrophic factor (CNTF), cardiotrophin-1 (CT-1), and hepatocyte growth factor (HGF), as follows: 1 ng/ml BDNF, 10 ng/ml GDNF, 10 ng/ml CNTF, 10 ng/ml CT-1, and 10 ng/ml HGF (Peprotech). At 24 h after plating, 2 μg/ml of aphidicolin (Sigma) was added to the culture medium and was maintained throughout the experiment.

### Calpeptin in vivo administration

Mice were individually housed in propylene cages (33 cm × 18 cm × 14 cm) at an ambient temperature of 22 ± 2 °C and relative humidity of 40 ± 10%. Breeder mice were provided with ad libitum water and rodent chow. Mice were maintained on a 12 h:12 h light:dark cycle (light period 07:30–19:30). SMNdelta7 and WT littermates were assigned by simple randomization to receive treatment or vehicle. Males and females were equally included in the study. Calpeptin (Calbiochem) was dissolved in DMSO (50 mM calpeptin) and injected at a dose of 6 μg per gram of weight in saline solution. Vehicle groups (Sham) received equal volumes of saline solution with the same amount of DMSO. No blinded administration was via subcutaneous (SC) injection in the interscapular region once a day starting from P0 with a polypropylene sterile syringe (Icogamma plus, 1 mL) and with a 30 G needle (BD Microlance). WT and mutant animals received treatment or vehicle to a maximum of postnatal day 8 (P8). Birth was defined as postnatal day 0 (P0) for the experiments.

### Human fibroblast cell lines culture

Human fibroblast cell lines were obtained from the Coriell Institute for Medical Research (Camden, NJ, USA). The Coriell Cell Repository maintains the consent and privacy of the donor samples. All the cell lines and culture protocols in the present study were carried out in accordance with the guidelines approved by institutional review boards at the Universitat de Lleida and IRBLleida research center. Two human fibroblast cell lines from patients with SMA (3813: GM03813, SMAII; and 9677: GM09677, SMAI) and one unaffected control (3814: GM03814, Control) were purchased and cultured following manufacturer instructions. Cells were maintained in minimum essential medium eagle (MEME) (Sigma) supplemented with non-inactivated fetal bovine serum (Gibco) (15% v/v), 0.5 M of l-glutamine (Gibco), 1% (v/v) of nonessential amino acids (Gibco), and 20 µg/ml Penicillin–Streptomycin (Gibco). Cells were subcultured every 3–4 days. For western blot analysis, cells were plated at a density of 3000–4000 cells/cm^2^ in 35 mm tissue culture dishes and maintained in supplemented MEME medium. After 2 days, total cell lysates were collected and submitted to western blot analysis.

### Differentiation of human-induced pluripotent stem cells (iPSCs) to MNs

The human iPSCs used in the present work were purchased from Coriell Institute for Medical Research. The control cell line was GM23411*B iPSC (healthy non-fetal tissue) and the SMA cell line was GM23240*B iPSC from a patient with SMA type II (*SMN2* 2 copies; delta exon7–8 in *SMN1*). Control and SMA cells were differentiated to MNs following the protocol described previously^24^ with minor modifications. Briefly, the human iPSCs were cultured on a layer of irradiated mouse embryonic fibroblasts (MEFs) (Gibco). To generate neuroepithelial cells (NEP), iPSCs were dissociated with Accutase (Gibco) following manufacturer indications and plated on Geltrex (Gibco)-coated plates in MEF-conditioned medium. After 24 h, fresh neuroepithelial induction medium (NEPIM: DM/F12:NBM 1:1 supplemented with B27, l-glutamine, NEAA, all from Gibco; 0.1 mM ascorbic acid, Sigma; 3 μM CHIR99021; 2 μM SB431512; 2 μM DMH1, all from Cayman) was added. Cells were maintained in culture for 6 days, changing the medium every other day, and dissociated with Accutase to generate MN progenitors (MNP). MNPs were expanded with NEPIM containing 0.1 μM retinoic acid (Sigma), 0.5 μM purmorphamine (Cayman), and 0.5 mM valproic acid (Sigma).

To induce MN differentiation, MNPs were detached with Accutase and cultured in suspension in MN induction medium (NEPIM plus 0.5 μM retinoic acid, 0.1 μM purmorphamine). Medium was changed every other day. After 6 days, neurospheres were dissociated with Accumax (Invitrogen) and plated on laminin-coated plates in MN maturation medium (MN induction medium supplemented with 0.1 μM Compound E, Abcam; 20 ng/ml CNTF and 20 ng/ml insulin-like growth factor 1, IGF-1, Peprotech). For western blot analysis, cells were plated on laminin-coated four-well dishes at 60,000 cells/cm^2^ of density and maintained with MN maturation medium during 6 days. For immunofluorescence experiments, 15,000 cells were plated on laminin-coated 1 cm^2^ glass coverslips, maintained in the MN maturation medium, and fixed in 4% paraformaldehyde in phosphate-buffered saline (PBS).

### Immunofluorescence

Lumbar region 1 and lumbar region 2 (L1 and L2) segment of the spinal cords of calpeptin- or sham-treated SMNdelta7 and WT mice was dissected and fixed in 4% paraformaldehyde (Sigma) for 24 h. Three animals for each condition were used. Cryopreservation with 30% sucrose buffer was done 48 h before mounting segments in tissue freezing medium (TBS, Electron Microscopy Sciences), sectioned at 16 μm-thickness in a cryostat (Leica CM3000). Slices were permeabilized with 0.3% Triton X-100 and incubated for 1 h with 5% bovine serum albumin (BSA) in PBS. Cultured human and mice MNs were plated on glass coverslips and at the end of the treatment were fixed with 4% paraformaldehyde (Sigma) for 10 min followed by an additional min with cold methanol (Sigma), then incubated for 2 h with 5% BSA in PBS.

Primary antibody (anti-Islet1/2 antibody, 1:50, Cat. No. 39.4D5, Developmental Studies Hybridoma Bank; anti-βIIITubulin antibody, 1:400, Cat. No. 5568; anti-LC3 antibody, 1:100, Cat. No. 2775, both from Cell Signaling; anti-SMN antibody, 1:100, Cat. No. 610646, BD Biosciences; anti-HB9 antibody, 1:75, Cat. No. ab92606; anti-Calpastatin antibody, 1:100, Cat. No. ab28252; anti-ChAT antibody, 1:100, Cat. No. ab18736, all from Abcam; anti-Mu-Calpain antibody, 1:100, Cat. No. 9A4H8D3, Invitrogen) was diluted in 0.01% Triton X-100 and incubated overnight. After washing, the secondary antibody was added: anti-mouse ALEXA555 antibody, 1:400, Cat. No. A21422; anti-rabbit ALEXA488 antibody, 1:400, Cat. No. A11008, all from Invitrogen; anti-sheep ALEXA649, 1:400, Cat. No. 713-496-147; anti-mouse ALEXA488 antibody, 1:400, Cat. No. 715-546-150, both from Jackson ImmunoResearch. Counterstain was performed with NeuroTrace 530/615 Red Fluorescent Nissl Stain (1:150, Life Technologies). Hoechst (1:400, Sigma) to identify nuclear localization in MNs soma. Samples were mounted using Mowiol (Calbiochem) medium. Confocal images were obtained using an FV10i Olympus confocal microscope. Quantification of fluorescence was blinded performed using the NIH ImageJ software.

### Western blot analysis

Western blots were performed as previously described^22^. Spinal cord tissue samples (WT sham *n* = 5; WT calpeptin *n* = 4; mutSMA sham *n* = 6; and mutSMA calpeptin *n* = 4) were disaggregated using Direct Quant 100ST Buffer (DireCt Quant) and a G50 Tissue Grinder (Coyote Bioscience). Total cell lysates of cultured cells or tissue homogenates were resolved in sodium dodecyl sulfate polyacrylamide gels and transferred onto polyvinylidene difluoride Immobilon-P transfer membrane filters (Millipore) using an Amersham Biosciences semidry Trans-Blot. The membranes were blotted with anti-SMN antibody (1:5000, Cat. No. 610646, BD Biosciences), anti-Calpain-1 antibody (1:1000, Cat. No. CG1928, Biomol International Inc.), anti-Fodrin (Clone AA6) antibody (1:4000, Cat. No. FG6090, Biomol International Inc.), and anti-Calpastatin antibody (1:1000, Cat. No. AB28252, Abcam). To control the specific protein content per lane, membranes were reprobed with monoclonal anti-α-tubulin antibody (1:50,000, Cat. No. T5168, Sigma). Blots were developed using Luminata^™^ ForteWestern HRP Substrate (Millipore).

### Statistical analysis

All experiments were performed at least three independent times. For animal studies we selected the minimum number of mice to have enough subjects to accomplish experimental aims. No samples or animals were excluded from the analysis. Values were expressed as mean ± SEM. Statistical analyses were performed using GraphPad Prism (version 5) (GraphPad Software Inc.). Differences between groups were assessed with two-tailed Students *t* test or by one-way ANOVA with Bonferroni multiple comparisons post-test. Similar variances between the compared groups were assumed. Values were considered significant when *p* < 0.05.

## Results

### Calpain activity is increased in SMA cultured spinal cord MNs

**Fig. 1:**
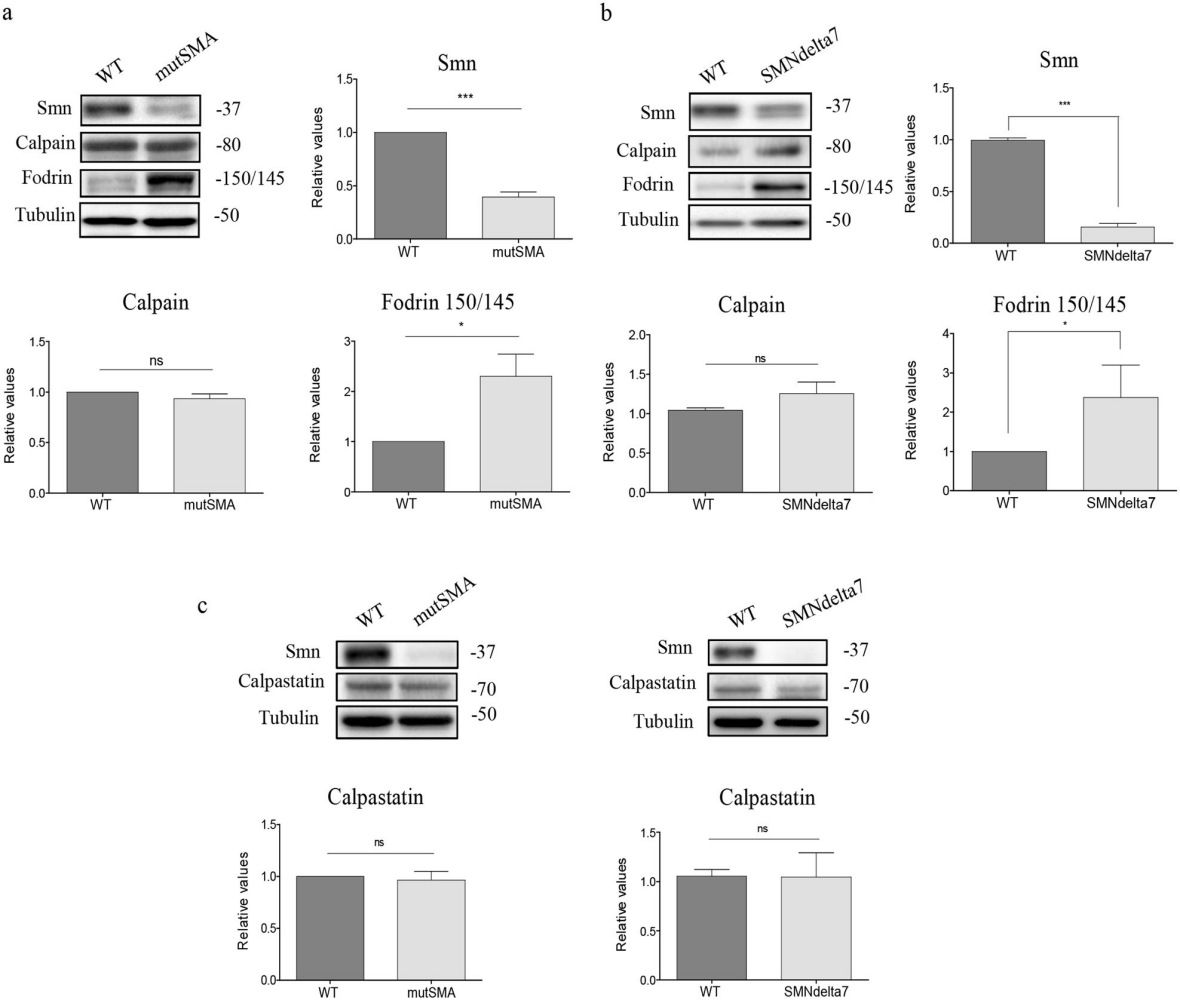
Fodrin 150/145 breakdown products are increased in cultured embryonic spinal cord MNs from SMA mice. Embryonic mouse motoneurons (MNs) were isolated from wild-type (WT: **a**–**c**) or mutSMA (**a**, **c**) or SMNdelta7 (**b**, **c**) genotyped embryos and cultured in the presence of a neurotrophic factors cocktail. Four days after plating, protein extracts were obtained and submitted to western blot analysis using anti-SMN antibody (**a**, **b**), anti-calpain 1 antibody (**a**, **b**), anti-fodrin antibody (**a**, **b**), or anti-calpastatin antibody (**c**). Membranes were reprobed with an anti-α-tubulin antibody. Graph values represent the expression of Smn, calpain, 150/145 fodrin product or calpastatin vs. α-tubulin and correspond to the quantification of 4 or 6 independent experiments. Asterisks indicate differences using Student *t* test (*p < 0.05; ***p < 0.0001).

In two SMA mouse models, in vivo treatment with the calpain inhibitor calpeptin has been shown to improve survival and motor phenotype^21^. In order to determine whether calpain pathway is altered in Smn-reduced MNs, mutSMA and SMNdelta7 E12.5 embryos were genotyped and the spinal cords of WT and mutant mice were dissected. MNs were isolated and cultured in the presence of a cocktail of neurotrophic factors. After 4 days, in vitro protein extracts were collected and submitted to western blot analysis using anti-SMN antibody, anti-α-fodrin antibody, anti-Calpain-1 antibody, or anti-calpastatin antibody. As expected, Smn protein level was significantly reduced in total cell lysates of cultured MNs from both SMA models (mutSMA, 0.39 ± 0.04, *p* < 0.0001; SMNdelta7, 0.16 ± 0.03, *p* < 0.0001), compared to the WT controls (Fig. 1). Degradation of α-fodrin to the 150/145 kDa-specific fodrin breakdown products is a well characterized method to evaluate calpain activation by western blot analysis^25^. Therefore, an anti-α-fodrin antibody was used to measure α-fodrin fragments in order to define calpain activation in WT and SMA samples. The levels of 150/145 kDa product in protein extracts obtained from mutSMA (2.30 ± 0.43, *p* = 0.014) (Fig. 1a) and SMNdelta7 (2.37 ± 0.8, *p* = 0.029) (Fig. 1b) cultured MNs were significantly increased compared to the respective WT controls. However, calpain protein level did not differ significantly between SMA cultured MNs and WT controls (Fig. 1a, b).

Taken together, these results suggested an increase of calpain activity in cultured Smn-reduced MNs without an increase of calpain protein level. To elucidate whether increased calpain activity was caused by a reduction of calpastatin protein level, we evaluated the level of this endogenous calpain inhibitor by western blot analysis. We observed that calpastatin was not significantly modified in mutSMA and SMNdelta7 conditions (Fig. 1), suggesting that increased calpain activity in these cells is not caused by reduced levels of calpastatin.

### Calpain activity is increased in human SMA iPSCs differentiated MNs, but not in SMA fibroblasts

**Fig. 2:**
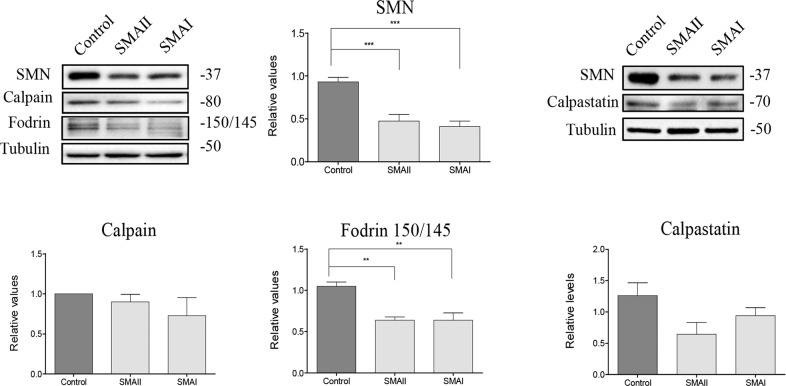
Analysis of fodrin, calpain, and calpastatin protein levels in cultured human SMA fibroblasts. Control (unaffected) and SMAII and SMAI patient fibroblast cell lines were plated in 35 mm culture dishes and maintained in the presence of supplemented MEME medium. Two days after plating, cell lysates were obtained and submitted to western blot analysis using anti-SMN antibody, anti-calpain 1 antibody, anti-fodrin antibody, or anti-calpastatin antibody. Membranes were reprobed with an anti-α-tubulin antibody. Graph values represent the expression of SMN, calpain, 150/145 fodrin product, or calpastatin vs. α-tubulin and correspond to the quantification of six independent experiments. Asterisks indicate differences using one-way ANOVA with Bonferroni multiple comparisons post-test (**p < 0.0003; ***p < 0.0001).

**Fig. 3:**
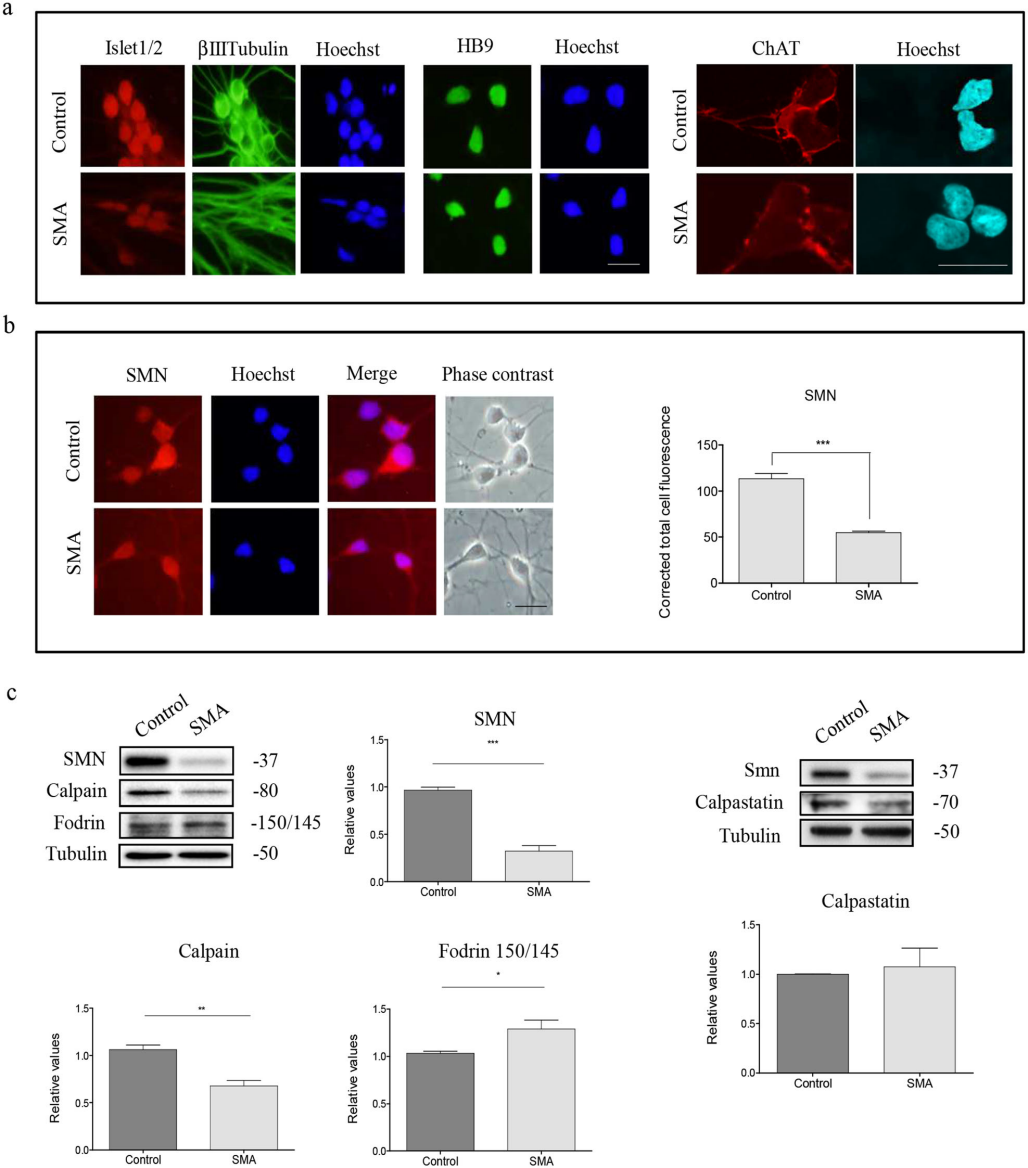
Calpain protein levels are reduced in human SMA iPSC differentiated MNs. **a** Representative immunofluorescence images of 3-day differentiated motoneurons (MNs) in Control and spinal muscular atrophy (SMA), showing Islet 1/2 (red), βIIITubulin (green left section), Hoechst (blue), and HB9 (green right section). **b** Representative immunofluorescence images of Control and SMA MNs using an anti-SMN antibody and Hoechst staining after 3 days of culture. Graph represents the mean of relative SMN fluorescence (red) measured in cell soma, corresponding to the quantification of three independent experiments ± SEM. Asterisks indicate significant differences using Student *t* test (*p* < 0.0001). **c** Protein extracts of 3-day differentiated human MNs were submitted to western blot analysis and probed with anti-SMN antibody, anti-calpain 1 antibody, anti-fodrin antibody, or anti-calpastatin antibody. Membranes were reprobed with an anti-α-tubulin antibody, used as a loading control. Graphs represent the expression of SMN, calpain, 150/145 fodrin products or calpastatin, corresponding to the quantification of at least three independent experiments ± SEM. Asterisks indicate differences using Student *t* test (*p < 0.05; **p < 0.001; ***p < 0.0001). Scale bar, 20 μm.

To further evaluate changes of calpain pathway members in SMN-reduced cells, we next explored protein levels of calpain, calpastatin, and fodrin in two human SMA cellular models: fibroblasts and MNs differentiated from iPSC (Figs. 2 and 3).

Total protein cell lysates of 2-day cultured fibroblasts were submitted to western blot analysis using the following antibodies: anti-SMN antibody, anti-calpain antibody, anti-α-fodrin antibody, or anti-calpastatin antibody. Results showed that SMN protein level was significantly reduced in cell lysates from SMA fibroblasts (SMAII, 0.47 ± 0.07; and, SMAI, 0.41 ± 0.06, *p* < 0.0001) compared to the clinically unaffected control. In SMN-reduced fibroblasts we observed that 150/145 kDa α-fodrin degradation product was significantly decreased (SMAII, 0.63 ± 0.04; and, SMAI, 0.64 ± 0.09, *p* < 0.0003) compared to control condition, indicating that calpain activity was reduced in these cells. Protein level of calpain and calpastatin were not significantly modified in SMA fibroblasts compared to the control (Fig. 2).

Human SMA and control iPSC cells were in vitro differentiated to MNs. Immunofluorescence analysis showed that MN markers such as Islet 1/2, HB9, and ChAT besides the neuronal indicator βIIITubulin were expressed by these cells after 6 days in the presence of MN maturation medium, suggesting that human SMA and Control iPSC cells were properly differentiated to MNs (Fig. 3a) following the protocol described^24^. As expected, SMN level was reduced in SMA samples compared to Control. Six days after differentiation, cells were fixed and SMN immunostaining with anti-SMN antibody was performed. Using the NIH ImageJ software, quantification of average of fluorescence units showed that SMN was reduced in SMA (54.7 ± 1.8, number of cells = 76; *p* < 0.0001) MNs compared to Control (113.3 ± 5.7, number of cells = 90) (Fig. 3b). Western blot analysis of total protein lysates using an anti-SMN antibody demonstrated that SMN protein was significantly decreased in SMA (0.32 ± 0.06, *p* < 0.0001) samples compared to Control (Fig. 3c). When calpain, 150/145 kDa α-fodrin and calpastatin were analyzed in protein extracts of 6 days differentiated MNs, we observed the following results: (i) 150/145 kDa α-fodrin degradation product was significantly increased in SMA samples (1.29 ± 0.10, *p* = 0.0278) compared to the Control condition; (ii) calpain protein level was significantly reduced in SMA samples (0.68 ± 0.06, *p* = 0.0010) compared to the Control condition; and (iii) calpastatin protein levels were not significantly different between SMA and Control conditions (*p* = 0.720) (Fig. 3c). These results indicate an increase of calpain activity together with reduced levels of calpain protein in SMA human differentiated MNs.

### Calpeptin treatment increases Smn protein level and reduces α-fodrin degradation product in SMA mutant mice

**Fig. 4:**
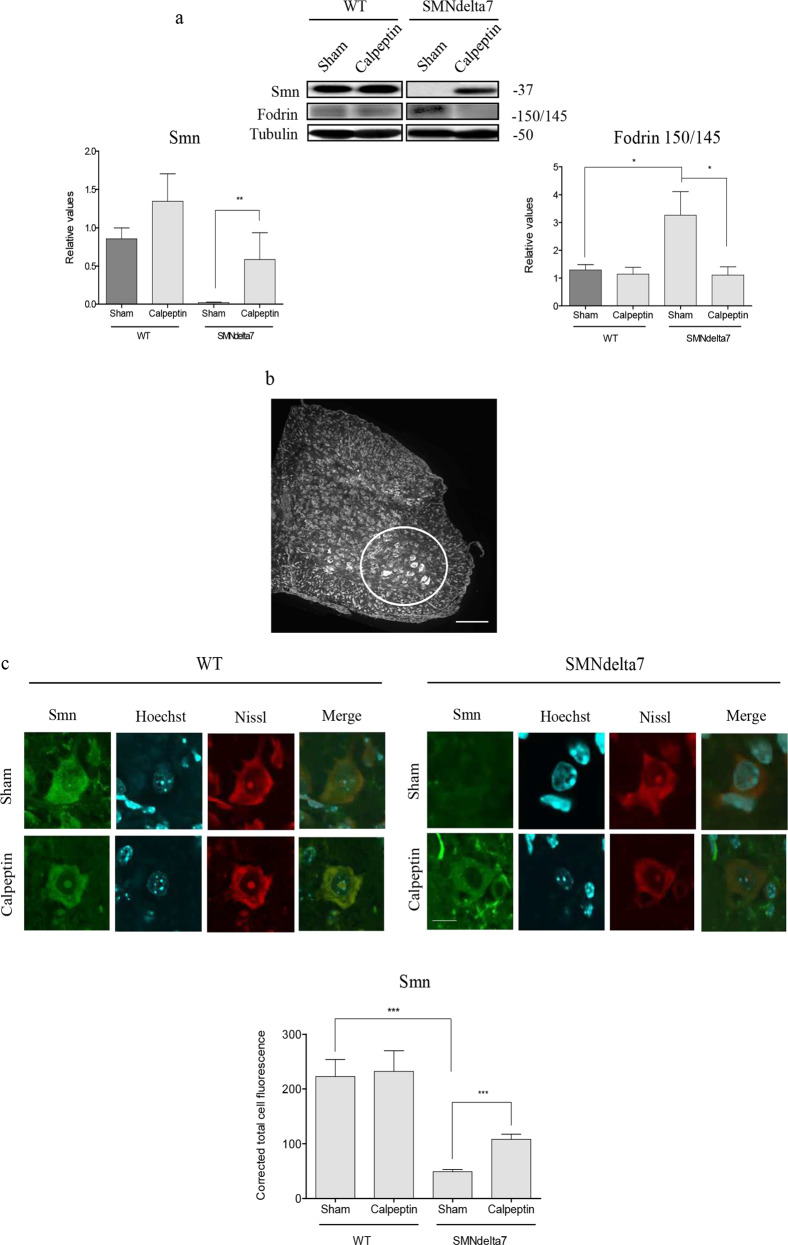
Effect of in vivo calpeptin treatment on Smn and fodrin levels in the spinal cord of SMNdelta7 mice. Wild-type (WT) and SMNdelta7 genotyped mice were daily treated with vehicle or 6 μg/g of calpeptin and sacrificed at 8 post-natal days. **a** Total cell lysates of spinal cords were submitted to western blot analysis using anti-SMN antibody or anti-fodrin antibody. Membranes were reprobed with an anti-α-tubulin antibody, used as a loading control. Graphs represent the expression of Smn or 150/145 fodrin products and correspond to the quantification of at least four treated animals from three independent experiments ± SEM. Asterisks indicate differences using Student *t* test (*p < 0.05; **p < 0.005). **b** Representative immunofluorescence image of lumbar spinal cord section using Nissl staining. Red circle indicates ventral MNs pool localization. Scale bar, 200 µm. **c** Representative immunofluorescence images of ventral horn spinal cord sections of WT and SMNdelta7 vehicle- or calpeptin-treated mice using an anti-SMN antibody (green) and Nissl staining (red). Scale bar, 10 μm. Nissl and Hoechst (blue) staining were used to identify MNs soma and nucleus, respectively. Graph represents the mean of relative Smn fluorescence measured in MNs soma, corresponding to the quantification of at least 50 neurons per condition from sections of minimum 4 treated animals ± SEM. Asterisks indicate significant differences using one-way ANOVA with Bonferroni multiple comparisons post-test (***p < 0.0001). Images were acquired with an FV10I confocal microscope (Olympus) using the ×60 objective and the same microscopy settings. Images were not submitted to any post-capture manipulation.

Calpeptin is a cell-permeable calpain inhibitor which increases Smn protein level in cultured isolated mice spinal cord MNs. In addition, in vivo calpeptin treatment extends SMA mice survival and improves motor function^21^. To further examine the cellular effect of in vivo calpeptin administration we started a treatment protocol using the FVB.Cg-Grm7Tg(SMN2)89Ahmb Smn1tm1Msd Tg (SMN2*delta7)4299Ahmb/J (SMNdelta7) mouse model. This severe SMA mouse model has a life expectancy of 10 days, and calpeptin treatment increases lifespan to 14 days^21^. SC injection of calpeptin (6 μg per gram of weight in saline solution) was administrated once a day starting from postnatal day 0 (P0) to postnatal day 8 (P8). Vehicle (Sham) and calpeptin treated mice were sacrificed at P8. In order to determine the level of Smn protein and calpain activation in spinal cord of treated animals, total protein extracts of lumbar fragments were obtained and submitted to western blot using anti-SMN antibody and anti-α-fodrin antibody. Results showed that Smn level was significantly increased in protein extracts from calpeptin-treated SMNdelta7 mice (0.58 ± 0.03, *p* = 0.0095; *n* = 4 treated animals) compared to the vehicle-treated control extracts (SMNdelta7 Sham) (Fig. 4a). Even it was observed a slightly increase of Smn in calpeptin-treated WT (1.34 ± 0.03; *n* = 4 treated animals), no significant differences were observed compared to the Sham control condition (0.85 ± 0.14, *p* = 0.32; *n* = 4 treated animals). We also analyzed the level of 150/145 kDa α-fodrin fragments in protein extracts of calpeptin-treated mice. As shown in Fig. 4a, 150/145 kDa α-fodrin fragment was significantly increased in SMNdelta7 Sham (3.26 ± 0.84) compared to WT Sham (1.29 ± 0.19, *p* = 0.021), indicating an increased calpain activity in SMNdelta7 spinal cord protein extracts. On the other hand, calpeptin-treated SMNdelta7 condition showed 150/145 kDa α-fodrin (1.11 ± 0.29) levels, which is not different to WT Sham controls, but it is significantly reduced compared to SMNdelta7 Sham (*p* = 0.031). Together these results suggest that SMNdelta7 spinal cord protein extracts show increased calpain activity which is prevented by SC injection of calpeptin.

In order to know whether Smn protein level is increased in lumbar spinal cord MNs soma of SMNdelta7 calpeptin-treated mice, we performed an immunofluorescence analysis using an anti-SMN antibody. Mice were subcutaneously treated daily with calpeptin from P0 and sacrificed at P8. Lumbar fragments of spinal cords of Sham- or calpeptin-treated WT and SMNdelta7 mice were dissected and cryostat sections of 16 μm-thickness were obtained. MNs were identified in spinal cord sections using Nissl stain, and Smn corrected total cell fluorescence was measured in Nissl-positive MNs. As predictable, quantification of the relative average of fluorescence units demonstrated that Smn level was significantly reduced in Sham-treated SMNdelta7 cells (49.1 ± 3.8, *p* < 0.0001) compared to the WT Sham control (222.8 ± 30.8). No significant differences of Smn fluorescence were observed in WT Sham- and WT calpeptin-treated cells (232.2 ± 38, *p* = 0.848) (Fig. 4b). However, calpeptin treatment increased significantly Smn relative fluorescence in SMNdelta7 MNs soma (108.2 ± 9.4, *p* < 0.0001) compared to sham-treated SMNdelta7, indicating that calpain inhibition increases Smn level in spinal cord MNs.

**Fig. 5:**
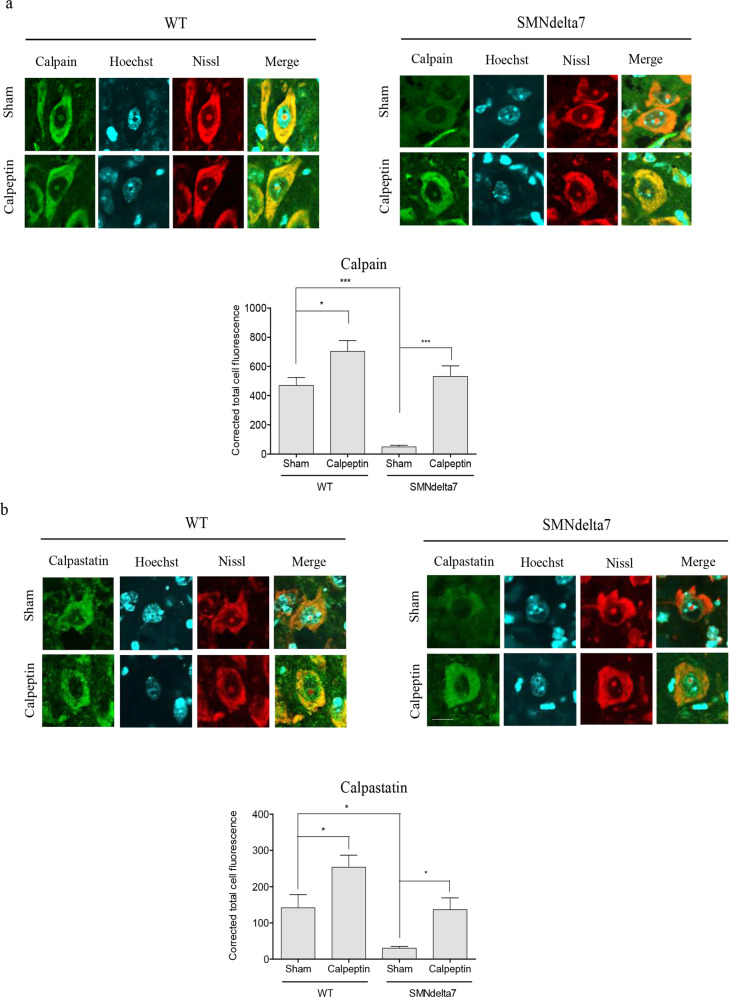
Calpeptin in vivo treatment increase calpain and calpastatin in SMA spinal cord MNs. Wild-type (WT) and SMNdelta7 genotyped mice were daily treated with vehicle (Sham) or 6 μg/g of calpeptin (Calpeptin) and sacrificed at 8 post-natal days. Representative immunofluorescence images of ventral horn spinal cord sections of WT and SMNdelta7 vehicle- or calpeptin-treated mice using anti-calpain 1 antibody (green) (**a**) or anti-calpastatin antibody (green) (**b**) and Nissl staining (red). Scale bar, 10 μm. Nissl and Hoechst (blue) staining were used to identify MNs soma and nucleus, respectively. Graph represents the mean of relative calpain (**a**) or calpastatin (**b**) fluorescence measured in MNs soma, corresponding to the quantification of at least 50 neurons per condition from sections of minimum 3 treated animals ± SEM. Asterisks in **a** and **b** indicate significant differences using one-way ANOVA with Bonferroni multiple comparisons post-test (*p < 0.05; **p < 0.001; ***p < 0.0001). Images were acquired with an FV10I confocal microscope (Olympus) using the ×60 objective and the same microscopy settings. Images were not submitted to any post-capture manipulation.

To further analyze the calpain pathway in spinal cord MNs of calpeptin-treated mice, we decided to measure by immunofluorescence the level of calpain and calpastatin in cell soma. To this end, lumbar spinal cord sections of 8 days sham- and calpeptin-treated WT or SMNdelta7 mice were processed for immunofluorescence using an anti-calpain antibody or an anti-calpastatin antibody. Results indicate that SMNdelta7 Sham-treated (47.87 ± 4.91) MNs showed significantly reduced levels of calpain compared to WT Sham-treated (469.4 ± 52.78, *p* < 0.0001) control condition. Calpeptin treatment significantly increases calpain level in both WT (702.7 ± 74.16) and SMNdelta7 (531.8 ± 71.62) conditions compared to WT (*p* < 0.05) and SMNdelta7 (*p* < 0.0001) Sham-treated control samples, respectively. This result indicated that calpeptin treatment regulates calpain protein level in spinal cord MNs soma. On the other hand, the analysis of calpastatin levels in the same experimental context showed a comparable profile to the one observed in calpain immunofluorescence measures. Sham-treated SMNdelta7 (30.2 ± 4.9) MNs exhibited decreased calpastatin levels compared to sham WT (141.6 ± 35.9, *p* < 0.05) controls (Fig. 5b). Calpeptin treatment increased calpastatin average fluorescence units in both WT (253.7 ± 32.9) and SMNdelta7 (137.0 ± 32.1) MNs soma compared to WT (*p* < 0.05) and SMNdelta7 (*p* < 0.05) sham-treated conditions, respectively. These results together suggest that in vivo calpain inhibition using calpeptin administration increases endogenous levels of calpain and calpastatin.

### Effect of calpeptin treatment on LC3 level in cultured SMA spinal cord MNs

**Fig. 6:**
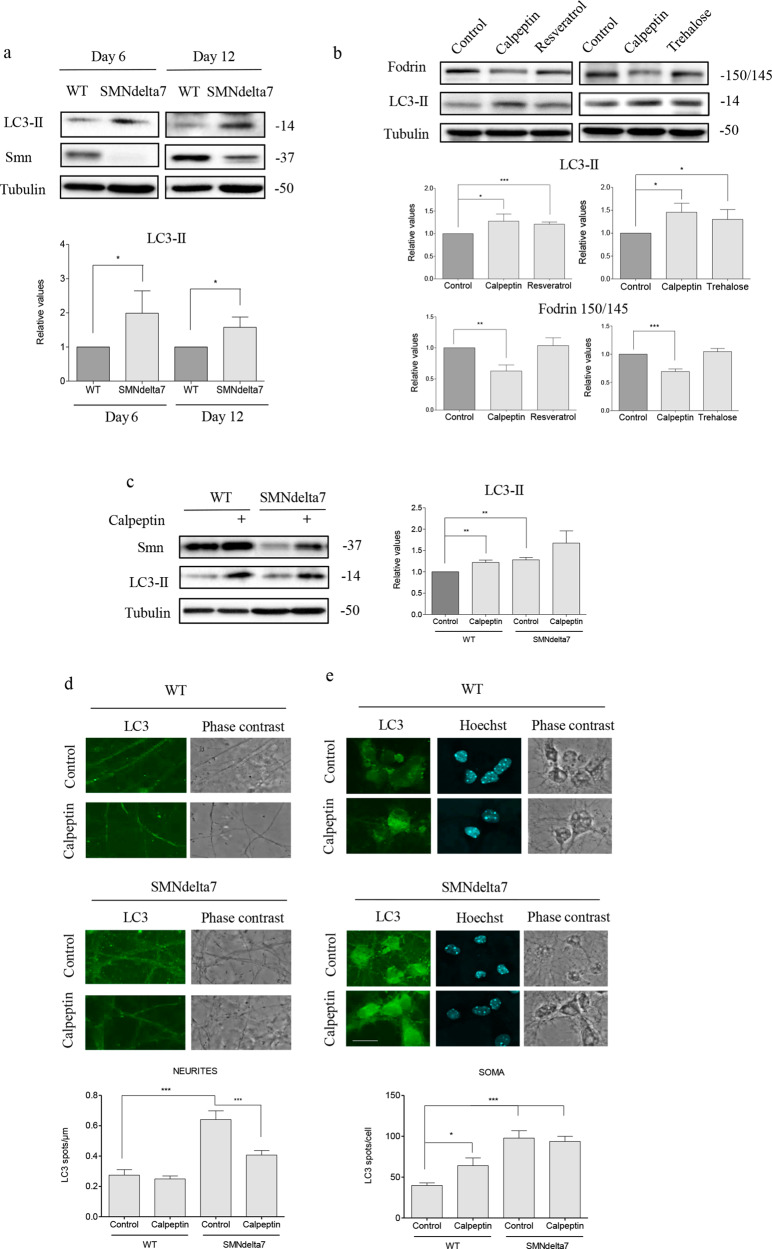
In vitro analysis of LC3 autophagosome marker in calpeptin treated cells: soma and neuritis. **a** WT and SMNdelta7 spinal cord motoneurons (MNs) were isolated and cultured in the presence of a neurotrophic factors cocktail. After 6 and 12 days in vitro protein extracts were obtained and submitted to western blot analysis using an anti-LC3 antibody. Membranes were reprobed with an anti-α-tubulin antibody, used as a loading control. Graph represents the expression of LC3-II corresponding to the quantification of three independent experiments ± SEM. Asterisks indicate differences using Student *t* test (*p < 0.05). **b** Isolated CD1 E12.5 mouse MNs were cultured in the presence of a neurotrophic factors cocktail. Six days after plating cells were left untreated or treated with 25 µM calpeptin or 50 nM resveratrol for 12 h; or with 25 µM calpeptin or 100 mM trehalose for 6 h. Total cell lysates were submitted to western blot using an anti-α-fodrin antibody or an anti-LC3 antibody. Membranes were reprobed with an anti-α-tubulin antibody, used as a loading control. Graphs represent the expression of LC3-II or 150/145 fodrin products corresponding to the quantification of 3 independent experiments ± SEM. Asterisks indicate differences using Student *t* test (*p < 0.05; **p < 0.005; ***p < 0.0001). **c** WT and SMNdelta7 MNs were cultured in the presence of a neurotrophic factors cocktail. Six days after plating cells were left untreated or treated with 25 µM calpeptin during 3 h. Cells lysates were obtained and submitted to western blot using an anti-SMN antibody or an anti-LC3 antibody. Membranes were reprobed with an anti-α-tubulin antibody, used as a loading control. Graphs represent the expression of LC3-II corresponding to the quantification of three independent experiments ± SEM. Asterisks indicate differences using Student *t* test (**p < 0.005). **d** WT and SMNdelta7 MNs were isolated and cultured during 6 days in the presence of a neurotrophic factors cocktail. Cells were treated with 25 μM calpeptin or left untreated during 3 h and immunofluorescence was performed using anti-LC3 antibody (green) (**d**, **e**). Representative confocal images of neurites (**d**) and soma (**e**) of immunostained MNs. Hoechst (blue) dye was used to identify MN nucleus. Graphs represent the mean of LC3 positive puncta measured in neurites (**d**) or soma (**e**) of wild-type (WT) and SMNdelta7 control or calpeptin treated MNs, corresponding to the quantification of tree independent experiments ± SEM. Asterisks indicate significant differences using one-way ANOVA with Bonferroni multiple comparisons post-test (*p < 0.05; ***p < 0.0001). Scale bar, 20 μm.

Autophagy is deregulated in SMA models^20,26^. Our previous studies demonstrated that the autophagosome marker LC3-II is increased in mice Smn-reduced spinal cord MNs in vivo and in vitro^27,28^. To corroborate autophagy alterations in the SMNdelta7 mouse model, we measured LC3-II protein level in cultured spinal cord MNs. WT and SMNdelta7 E12.5 embryos were genotyped and isolated MNs were cultured in the presence of the neurotrophic factors cocktail. After 6 or 12 days in culture WT and SMNdelta7 protein extracts were collected and submitted to western blot analysis using an anti-LC3 antibody. LC3-II protein level was significantly increased in total cell lysates of mutant MNs at 6 and 12 days in vitro (day 6: 1.98 ± 0.65, *p* = 0.04; day 12: 1.58 ± 0.30, *p* = 0.02) compared to the WT controls (Fig. 6a).

In many disease conditions and models calpains were shown to negatively regulate autophagy^14^. In this context, our next objective was to elucidate whether autophagy modulation can affect calpain activity in cultured MNs. To this end, 150/145 kDa α-fodrin degradation products were measured in the presence of the mTOR-dependent or the mTOR-independent autophagy inducers resveratrol^29^ and trehalose^30^, respectively. E12.5 CD1 mouse spinal cords were dissected and MNs were isolated and cultured during 6 days in the presence of neurotrophic factors. Cells were left untreated (Control) or treated with 25 µM calpeptin or 50 nM resveratrol for 12 h; or with 25 µM calpeptin or 100 mM trehalose for 6 h. After treatment, cell lysates were obtained and submitted to western blot using an anti-α-fodrin antibody or an anti-LC3 antibody. Results showed that calpeptin, resveratrol and trehalose treatments significantly increased LC3-II protein level (calpeptin 12 h: 1.27 ± 0.15, *p* = 0.03; calpeptin 6 h: 1.45 ± 0.19, *p* = 0.01; resveratrol 1.21 ± 0.05, *p* < 0.0001; trehalose 1.30 ± 0.21, *p* = 0.01) compared to untreated controls (Fig. 6b). As expected, calpeptin treatment significantly reduced 150/145 kDa α-fodrin fragments (12 h: 0.62 ± 0.09, *p* = 0.001; 6 h: 0.69 ± 0.04, *p* < 0.0001) compared to the untreated controls. Nevertheless, resveratrol and trehalose addition did not significantly modify α-fodrin fragments level (1.03 ± 0.12, *p* = 0.76 and 1.04 ± 0.05, *p* = 0.54, respectively) compared to untreated controls (Fig. 6b). These results suggest that the autophagy inducers resveratrol and trehalose did not modulate calpain activity in cultured spinal cord MNs.

We next analyzed whether calpeptin treatment regulate LC3 level in Smn-reduced MNs. SMNdelta7 E12.5 embryos were genotyped and the spinal cords of WT and mutant mice were dissected. MNs were isolated and cultured in the presence of neurotrophic factors. After 6 days in vitro, cells were treated with 25 µM calpeptin for 3 h and protein extracts were collected and submitted to western blot analysis using an anti-SMN antibody or an anti-LC3 antibody. LC3-II protein level was significantly increased in total cell lysates of mutant cultured MNs (SMNdelta7, 1.28 ± 0.05, *p* = 0.0024) compared to the WT controls (Fig. 6c). Addition of calpeptin to the culture medium significantly increased LC3-II protein in WT cultures (1.29 ± 0.05, *p* = 0.0034) compared to untreated WT controls. However, even LC3-II level was increased in calpeptin-treated SMNdelta7 condition (1.67 ± 0.28, *p* = 0.17), the differences were not statistically significant compared to untreated SMNdelta7 controls (Fig. 6c). Since MNs are highly polarized cells, we decided to examine LC3 protein in cell soma and neurites by immunofluorescence in calpeptin-treated and untreated conditions. To this end, isolated MNs from WT and SMNdelta7 were cultured in the presence of neurotrophic factors. Six days after plating cells were treated with 25 μM calpeptin during 3 h. Cultures were fixed and submitted to immunofluorescence using an anti-LC3 antibody. The number of fluorescent LC3 puncta was quantified using the NIH ImageJ software^28^. As expected, results shown in Fig. 6 exhibit a significant increase of LC3 in neurites and soma of SMNdelta7 (SMNdelta7 Control, neurites: 0.64 ± 0.05 spots; soma: 97.71 ± 9.1 spots) conditions compared to the WT (WT Control, neurites: 0.27 ± 0.03 spots, *p* < 0.0001; soma: 39.72 ± 3.24, *p* < 0.0001) controls. Calpeptin treatment significantly increases LC3 spots in WT MNs soma (64.1 ± 9.34, *p* < 0.05) compared to non-treated control cells, but a similar effect was not observed in neurites. However, calpeptin treatment clearly prevents LC3 increase in neurites of SMNdelta7 MNs (SMNdelta7 Calpeptin 0.4 ± 0.003, *p* < 0.0001) compared to the SMNdelta7 control cultures, indicating that calpeptin treatment reduced autophagosomes in neurites of SMA mice. We did not observe the same effect in MN soma. When fluorescent LC3 spots were measured in calpeptin-treated SMNdelta7 cell soma (93.67 ± 6.3), there were no significant differences compared to control non-treated SMNdelta7 cells. This result suggests that calpain inhibition increases LC3 spots in MN soma and prevents autophagosome accumulation in neurites.

## Discussion

The molecular mechanisms leading to MN degeneration in SMA disease are poorly understood. SMN protein reduction causes cellular changes which directly or indirectly provoke the deregulation of several processes involved in cell homeostasis, including autophagy and apoptosis^31^. Nonetheless, intracellular pathways related to neurotrophic communication or calcium regulation may be impaired in SMA pathology and contribute to modifying the evolution of the disease^32^. In this context, we have previously demonstrated that treating SMA mice with the calpain inhibitor calpeptin had a positive effect on survival and motor function^21^. The present work found an increase of calpain activity in cultured SMA spinal cord MNs and human differentiated SMA MNs. Calpain is a calcium-regulated protease implicated in neuronal injury, neurodegenerative disorders, and neuronal aging processes. Its overactivation has been detected in many neurodegenerative disorders, including amyotrophic lateral sclerosis^33^. Cellular functions of calpains are related to the regulation of the proteolysis of many substrates, including enzymes and structural proteins. This implies the control of several primary cell functions such as motility or gene expression^34^. Degradation of 270 kDa α-fodrin (also called αII-spectrin) to the 150/145 kDa-specific fodrin breakdown product has been attributed to the activation of calpain^25^. We observed an increase of these calpain-specific fodrin products in mice cultured spinal cord MNs and differentiated human SMA MNs, but not in human SMA cultured fibroblasts, suggesting an increase of calpain activity in SMN-reduced MNs. This increase of calpain activity was not the consequence of an increase of calpain protein, because no increase from basal level occurred in cultured mice MNs, and a decrease was observed in human differentiated SMA MNs and in MNs soma of SMNdelta7 mice.

Reports of the activation mechanisms of calpains have been controversial. The calcium concentrations necessary for activation of calpains are in the micro- to millimolar range, which is rather beyond the nanomolar calcium levels in cells under normal physiological conditions. Nevertheless, this apparent contradiction can be resolved because the cellular microenvironment may provide the sufficient calcium concentration^35^. Calpain activity can be compartmentalized and calcium changes localized in some neuronal sections in SMA may contribute to the increase of calpain activity in these particular spaces^36,37^.

However, damaging calpain overactivation has been detected in other neurodegenerative disorders, such as Alzheimer disease, amyotrophic lateral sclerosis, Parkinson disease, or the group of polyQ disorders^14^. This calpain overactivation can be caused by an increase of intracellular calcium concentration or by a reduction of the natural endogenous inhibitor calpastatin. Calpains form a complex with calpastatin, the only known endogenous protein inhibitor of calpain activity in cells^34^. Depletion of endogenous calpastatin has been described in mouse models of several neurodegenerative disorders^38–40^. Likewise, calpastatin reduction and increased calpain activity have been related to biochemical changes related to the breakdown of cytoskeletal proteins and activation of cdk5 and caspase-3, leading to MN degeneration in hSOD1^G93A^ mice^16^. Western blot analysis of calpastatin levels in cultured SMA MNs showed no changes in this protein in these cells; however, when immunofluorescence experiments were performed in spinal cords of P8 SMNdelta7 mice, we observed calpastatin reduction in MNs soma. In vivo calpeptin treatment prevents calpastatin reduction, which suggests that calpain activity can also regulate the members of the pathway. In this context, we observed that basal levels of calpain were increased by calpeptin treatment, reinforcing the hypothesis that calpain activity regulates calpastatin and calpain protein levels in spinal cord MNs. Interestingly, in vivo calpeptin treatment also increased SMN protein in spinal cord extracts and in MNs soma. It is well-known that SMN is a proteolytic target of calpain and its activity modulates SMN level^21,28,41,42^. Therefore, our results strongly suggest that the in vivo beneficial effect observed in calpeptin-treated SMA mice^21^ can be mediated to some extent by SMN protein increase in MNs.

Calpains are considered as modulator proteases that can regulate protein functions, and therefore various cellular pathways, including autophagy. In many disease environments and models, calpains are known to negatively regulate autophagy. Consequently, enhanced calpain activation can contribute to the compromised activation of this degradation pathway. Various studies have found that the impact of calpains on autophagy occurs on multiple levels, such as reduction of autophagy initiation or switching from autophagy to apoptosis^14^. Several neurodegenerative disorders exhibit an overactivation of calpains and disturbances of the autophagic pathway. In SMA disease, autophagy pathway is deregulated^26–28^ and inhibition of autophagy delays MN degeneration and extends lifespan of SMA mice^20^. From the results presented in these previous reports it can be suggested that LC3-II autophagosome marker is augmented in SMN-reduced MNs. In the present work we show that LC3-II protein level is increased in the SMNdelta7 mutant isolated MNs compared to the WT controls. It has been previously described that calpeptin treatment increases the number of autophagosomes-like structures in COS-7 cells^43^; accordingly, calpeptin treatment increased LC3-II protein level in WT conditions, but no significant differences were observed in SMNdelta7 cultures. By immunofluorescence, we showed that calpeptin treatment increased LC3 spots in MN somas of WT cells. However, we did not observe the same effect in neurites: LC3 spots in WT neurites did not increase after calpeptin treatment. Interestingly, calpeptin treatment prevented LC3 increase in neurites in SMA cells, but not in cell soma. Accumulation of autophagosomes in axons can perturb microtubule-associated axonal transport, which can be linked to neurodegeneration affecting autophagic flux. Our results suggest that calpeptin treatment may regulate autophagosome accumulation in neurites. This effect would prevent the neurite collapse that can lead to MN degeneration.

In conclusion, calpain pathway is an intracellular proteolytic mechanism involved in the development of several neurodegenerative disorders. The present study found an increase of calpain activity in SMN-reduced cells. We suggest that the positive modifier effect of in vivo calpeptin treatment on SMA mice survival and motor phenotype could be the result of SMN protein and LC3 autophagosome protein regulation in SMA MNs and could offer a viable therapeutic approach. Although infants with SMA appear to have a genetic defect in common, the clinical course of this disease shows high variability. The analysis of new SMA modifiers belonging to several intracellular regulatory pathways which contribute differently to disease progression may lead to the discovery of new treatment strategies for this devastating condition.
